# Navigating a complex dance: the interplay between RNA-binding proteins and T cells in oral epithelial plasticity

**DOI:** 10.1097/IN9.0000000000000054

**Published:** 2025-01-10

**Authors:** Anitha Vijayakumar, Sekar Vasudevan, Samu John, Michelle A. Ozbun, Eric Bartee, Viswanathan Palanisamy

**Affiliations:** 1 Division of Molecular Medicine, Department of Internal Medicine, University of New Mexico, Albuquerque, NM, USA

**Keywords:** oral squamous cell carcinoma, post-transcriptional gene regulation, RNA-binding protein, post-translational protein modification, immune cells and tumor microenvironment

## Abstract

The oral epithelium, a dynamic interface constantly facing environmental challenges, relies on intricate molecular pathways to maintain its homeostasis. This comprehensive review delves into the nuanced interplay between T-lymphocytic cells (T cells) and RNA-binding proteins (RBPs) within the oral epithelium, elucidating their roles in orchestrating immune responses and influencing tissue plasticity. By synthesizing current knowledge, we aim to unravel the molecular intricacies that govern this interplay, with a focus on potential therapeutic implications for oral health and diseases. Understanding the regulatory networks shaped by T cells and RBPs in the oral epithelial microenvironment holds promise for innovative strategies in managing conditions associated with epithelial dysfunction.

## 1. Introduction

Oral epithelial plasticity, which refers to the capacity of oral epithelial cells to transition between various states, is essential for the maintenance of a healthy oral cavity. Epithelial plasticity plays a crucial role in the processes of wound healing, tissue regeneration, and immunological response ^[1,2]^. The process of epithelial biogenesis, which involves the continuous regeneration of the oral lining, depends on a careful equilibrium between the immune system and gene regulation by RNA-binding proteins (RBPs) ^[3]^. The T cells play a crucial role in protecting the oral cavity from pathogens and regulating inflammation, while RBPs are responsible for coordinating gene expression and governing processes such as cell proliferation, differentiation, and tissue integrity ^[4]^. The two systems interact in a complex manner, where immune cells are influenced by RBPs, which regulate the immunological response. More so, the disruption of this process can contribute to the emergence and advancement of oral pathogenesis, including cancer, emphasizing the necessity to comprehend the fundamental mechanisms involved in this process. Therefore, understanding this interaction has significant promise for creating innovative therapeutic approaches for a variety of oral conditions, such as chronic inflammation and oral cancer.

## 2. RNA-binding proteins: architects of transcriptome regulation

The regulation of gene expression in the oral epithelium is a highly regulated process, where RBPs play a crucial role in controlling intricate transcriptome regulation. RBPs have long been acknowledged for their involvement in post-transcriptional gene regulation. They regulate RNA molecules, hence influencing gene expression patterns and controlling a range of biological activities such as splicing, stability, localization, and translation ^[5–7]^. Additionally, RBPs are involved in the regulation of long noncoding RNA (lncRNA), which affects chromatin structure and remodeling, histone changes, and DNA methylation ^[8]^. This emphasizes the complex nature of transcriptional control mediated by RBPs. Disruption of these processes is linked to many different disorders, making RBPs prospective targets for therapeutic intervention in conditions defined by abnormal gene expression. Studies have shown that RBPs have a vital role in regulating several stages of the metastatic cascade of oral squamous cell carcinoma (OSCC) within the setting of oral epithelial homeostasis. For example, the RNA editing protein adenosine deaminase acting on RNA (ADAR)-1 promotes migration and invasion, as well as stimulating squamous cell carcinoma cell growth, proliferation, and stemness ^[9]^. The RNA helicase DDX3 has been associated with metastasis, motility, and cell adhesion in OSCC by enhancing the translation of certain mRNAs ^[10,11]^. Recent findings have revealed that the renowned RNA-binding protein human antigen R protein (HuR) regulates the expression of crucial proteins associated with OSCC, hence facilitating the progression of tumor development and invasion. Our recent findings have demonstrated that HuR has a role in T cell reprogramming that enhances the development of OSCC ^[12,13]^. Epithelial splicing regulatory protein (ESRP)1 and ESRP2, which regulate alternative splicing, have been shown to influence the expression of crucial genes involved in epithelial-mesenchymal transition (EMT). This contribution of ESRP1 and ESRP2 has been observed in promoting cancer invasion and metastasis, as indicated by studies by Yae et al ^[14]^, and Horiguchi et al ^[15]^,. In a similar manner, IGF2BP3, a constituent of the IGF2BP family, regulates the transcription of genes that are linked to the spread of cancer and unfavorable outcomes in OSCC. Epiregulin (EREG) has been identified as a potential target for antimetastatic therapy due to its role as a functional mediator of IGF2BP2-regulated EMT and cell invasion in oral cancer ^[16,17]^. Gabanella et al ^[18]^, demonstrated the protein-protein interaction between survival motor neuron (SMN) protein with epidermal growth factor receptor (EGFR) to regulate E-cadherin influencing EMT in head and neck squamous cell carcinoma (HNSCC). Further, in 2017, study investigated the oncogenic functions of KH-type splicing regulatory protein (KHSRP) in esophageal squamous cell carcinoma (ESCC). It found that KHSRP expression is elevated in ESCC tumors, and its knockdown inhibits cell growth, migration, invasion, and miRNA maturation ^[19]^. Recently, we have demonstrated that the Fragile-X metal retardation protein, FXR1 also plays a role in altering the expression levels of proteins involved in EMT in OSCC ^[20]^. These investigations have demonstrated that RBPs play a crucial role in the progression of oral cancer by changing the homeostasis of the epithelial cells.

## 3. Dynamic tumor microenvironment in oral epithelium

Recent studies have shown the significant role of the tumor immune microenvironment (TIME), consisting of tumor-infiltrating lymphocytes (TILs) and tumor-associated macrophages (TAMs), in the progression of various oral pathological diseases, including oral tumors ^[21]^. TILs, specifically CD8 T cells, have a function that suppresses tumor growth ^[21]^. On the other hand, TAMs have various roles depending on their activation level. Activated T cells play a vital role in the adaptive immune system by identifying and eradicating foreign intruders, such as cancer cells. They accomplish this through many ways, such as directly eliminating target cells and releasing immunomodulatory chemicals ^[22]^.

TIME is the complex organization of noncancerous cells and extracellular matrix components that surround solid tumors. The malignancy of the behavior is ultimately defined by the reciprocal interactions between the tumor and TIME ^[23]^. The oral epithelium has a varied population of T lymphocytes, including both resident and infiltrating subsets ^[24]^. The different types of T cells, such as CD4^+^ and CD8^+^ T cells, have specific activities in monitoring and controlling the immune response as well as various functions in the oral epithelium ^[25]^. CD8^+^ T cells, also known as cytotoxic T cells, possess an impressive capacity to identify and eradicate infected or abnormal cells, therefore playing a crucial role in defending against infections and preserving tissue health ^[26]^. CD4^+^ T cells coordinate immune responses by assisting other immune cells, regulating the activation of B cells, and adjusting the activities of other T cells ^[27]^. The oral cavity, a gateway to the gastrointestinal and respiratory tracts, is exposed to a multitude of bacteria ^[28]^. T cells in the oral epithelium serve as the first line of defense against microbial threats by identifying antigens presented by nearby antigen-presenting cells ^[29]^. This recognition initiates a series of immunological reactions, resulting in the stimulation and mobilization of T cells to areas of infection or tissue injury.

## 4. Regulation of oral epithelial homeostasis by T cells

Gaining insight into the processes that establish and sustain homeostasis in the oral mucosa is key for human well-being. T cells actively contribute to the maintenance of oral epithelial homeostasis and in fighting against bacterial and viral infections. Various T cells with regulatory function exist, however, the predominant and significant regulatory T cells (Tregs) in the oral mucosa are CD4^+^ T cells that express CD25 and the master transcription factor FoxP3 ^[30]^. Treg cells release the cytokines interleukin (IL)-10, transforming growth factor (TGF)-β, and IL-35 ^[31]^. These cytokines contribute to immune suppression by restricting the production and release of pro-inflammatory proteins, reducing the expression of major histocompatability complex-II (MHCII) and co-stimulatory molecules, and decreasing T cell proliferation ^[32,33]^. T lymphocytes regulate the differentiation, proliferation, and repair mechanisms of epithelial cells through intricate molecular signaling. Due to its constant exposure to the environment, the oral mucosa is a location with significant immunological activity ^[22]^. Historically, T cells were believed to be the primary regulators of immunological reactions. In addition, Treg cells can impact the function of dentritic cells (DCs) by the interaction of cytotoxic T-lymphocyte antigen 4 with CD80/CD86, which competes with CD28 co-stimulation. Another mechanism involves the binding of lymphocyte activation gene 3 to MHCII, which interferes with antigen presentation function ^[34]^. Therefore, this reciprocal exchange of information between T cells and epithelial cells influences the overall structure of the tissue and maintains a careful equilibrium between immune protection and tissue regeneration. The existence of memory T cells in the oral epithelium confers a tactical benefit for swift and specific reactions upon encountering familiar antigens again. The presence of this reservoir of T cells enhances immune surveillance, providing defense against recurrent infections and aiding in the development of long-lasting oral immunological memory. Malfunctions in T cell activities within the oral epithelium can result in several clinical diseases. T cell responses have a crucial role in maintaining oral health, as evidenced by the changes observed in chronic inflammatory illnesses, autoimmune disorders, and oral malignancies. Exploring the intricacies of T cell dysregulation in particular oral illnesses shows potential for creating focused treatment approaches.

## 5. The interplay

**Figure 1.**
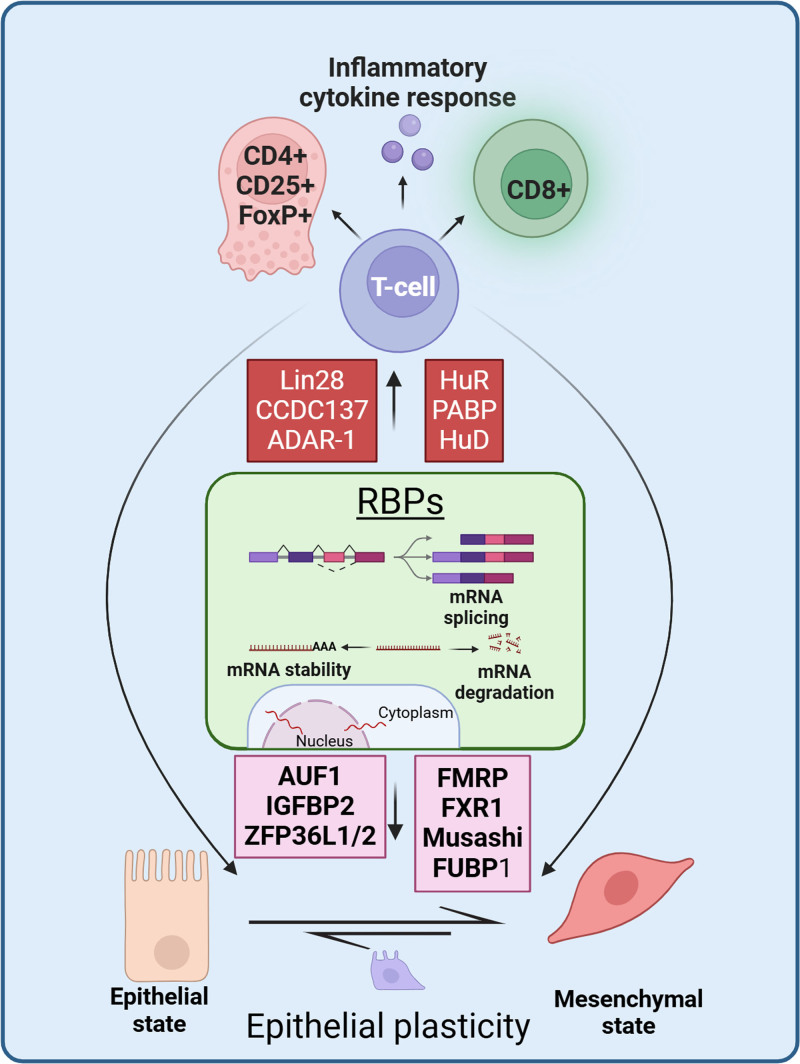
Model of the interplay between RBPs and the immune response. The schematic illustrates RBPs role in post-transcriptional regulation, including mRNA stability, splicing, translation, and localization. Key RBPs are depicted interacting with genes involved in immune response, and epithelial plasticity. RBP, RNA-binding proteins.

The intricate interaction between RBPs and T cells in modulating oral epithelial plasticity is multifaceted:

- T cells have the ability to control the expression of RBPs. Research indicates that some subsets of T cells, such as Tregs, can impact the expression of RBPs by releasing cytokines and other signaling molecules ^[35]^. This can result in changes in the activity of epithelial cells and contribute to the formation of a microenvironment that promotes tumor growth.
- RBPs have the ability to alter T cell activity and impact T cell function ^[35]^. RBPs have the ability to control the expression of genes related to T cell activation through m6A methylation ^[36]^. This can affect their capacity to generate a potent immune response against infections or cancer cells (Figure 1).
- Manipulating the activity of RBPs that support the epithelial phenotype could potentially reverse the process of EMT associated with the progression of oral cancer ^[37]^. This involves restoring epithelial differentiation by modulating RBP activity ^[37]^. These EMT processes are also governed by T cell cross-reactivity and alter tumor microenvironment ^[37]^.

Recent research has revealed the intricate network of interactions occurring in the oral epithelium. There is increasing evidence that T cells and RBPs play crucial roles in influencing immune responses and tissue plasticity ^[22]^. This artice seeks to thoroughly examine the interaction between these two elements, highlighting how they mutually affect each other in the specific environment of the mouth. Interestingly, several RBPs, such as HuR, have been identified as important regulators of post-transcriptional processes in oral epithelial cells ^[12,38,39]^. HuR, which plays a function in stabilizing, localizing, and translating mRNA, forms an intricate regulatory network that affects the expression of genes essential for maintaining epithelial homeostasis. The interaction between T cells and RBPs goes beyond typical immune responses, affecting the patterns of gene expression that determine the fate and adaptability of epithelial cells. Comprehending these molecular interactions not only provides insight into essential biological processes inside oral epithelium but also has significant implications for illnesses ranging from persistent inflammatory disorders to the complex dynamics of oral malignancies. This study comprehensively analyzes existing research to offer a thorough investigation of the interconnected functions of T cells and RBPs in oral epithelial cells. Our goal is to understand the regulatory mechanisms that control the interaction between oral health and disease. By doing so, we want to build the groundwork for future research and develop new therapeutic solutions to address the complex difficulties in oral health.

Table 1 provides an overview of various RNA-binding proteins and their functions in transcriptional regulation, showcasing the diversity of RBPs involved in influencing gene expression dynamics.

**Table 1.** An overview of various RNA-binding proteins and their functions in transcriptional regulation.

RNA-binding protein	Function in oral epithelium/post-transcriptional regulation	Cell type	References
HuR (ELAV1)	Stabilizes mRNA, modulating immune responses and epithelial cell fate decisions	Epithelial, mesenchymal, immune, adipocytes, neuronal, germ cells, muscle	^[12,13]^
PTB/PTBP1/hnRNP1	Influences alternative splicing, shaping T cell responses within the oral microenvironment	Neuronal, Immune cells	^[40]^
AUF1	Modulates mRNA stability and translation, impacting T cell function	Immune, endothelial, neuronal	^[41]^
TIA-1	Stress granule formation, alternative splicing, and mRNA translation	Neuronal, germ cells, Langerhans cells	^[42,43]^
FUS	Involved in transcriptional elongation and interacts with RNA polymerase II	Epithelial, germ, glial, neuronal, blood& immune, skeletal myocyte, endocrine, ductal	^[44]^
IGF2BP (eg,IGF2BP1, IGF2BP2, IGF2BP3)	Regulates mRNA stability and translational control, influencing gene expression	Germ cells, trophoblast, smooth muscle, fibroblast, blood& immune, specialized epithelium	^[45,46]^
LIN28	Plays roles in embryonic development and pluripotency; interacts with chromatin modifiers and transcription factors	Specialized epithelium, mesenchymal, germ cells, blood& immune	^[47]^
FMRP	Translation repression, neuronal development, and synaptic plasticity	Myocytes, neuronal, epithelial, muscle, immune, glial, endocrine, germ cells	^[48]^
PABP (PABPC1)	mRNA stability, translation initiation, and polyadenylation	Blood immune, germ cells, epithelial, endocrine, neuronal, glial, epithelial, mesenchymal, muscle	^[49]^
HuD (ELAVL4)	Neuronal development, mRNA stability, and alternative splicing	Neuronal, glial, endocrine, germ cells	^[50,51]^
Musashi1 (MSI-1)	Stem cell proliferation and cancer cell promotion	Neuronal, epithelial, glial, muscle	^[52]^
CCDC137	AKT signaling and hepatocellular carcinoma	Neuronal, epithelial, mesenchymal, blood& immune, endothelial, muscle, glial, endocrine, adipocytes, melanocyte, germ cells	^[53]^
ADAR-1	Growth, colony formation, migration/invasion, stemness	Neuronal, epithelial, mesenchymal, blood& immune, endothelial, muscle, glial, endocrine, adipocytes, melanocyte, germ cells	^[54]^
ZFP36L1 and ZFP36L2	Airway epithelium and subcellular mislocalization contribute to changes in mRNA expression and cytoplasmic fate	epithelial, mesenchymal, blood& immune, endothelial, muscle, glial, endocrine, adipocytes, melanocyte, germ cells	^[55]^
FUSE binding protein 1 (Fubp1)	14 revealed changes in the localization patterns oc-Myc and cell proliferation in epithelium and mesenchyme, related with altered tooth morphogenesis	Neuronal, epithelial, mesenchymal, blood& immune, endothelial, muscle, glial, endocrine, adipocytes, melanocyte, germ cells	^[56]^

ADAR, adenosine deaminase acting on RNA; HuR, Human antigen R protein.

## 6. HuR: a master regulator of oral health

The HuR, sometimes referred to as ELAVL1, is a well-studied RBP that is widely present in all tissues and is important for regulating gene expression after transcription. It plays a role in multiple cellular processes, such as cell growth, specialization, and reaction to stress ^[12]^. HuR has been discovered to play important roles in the immune system and oral epithelium ^[13]^. The interaction between HuR’s roles in the immune system and oral epithelium is intricate. HuR interacts with U- and AU-rich RNA motifs and undergoes nucleocytoplasmic shuttling, thereby modulating the maturation, processing, and stability of both coding and noncoding RNAs. The transportation is facilitated by signal-triggered interactions with nuclear export/import adaptors. HuR has the ability to modify the fate of the mRNAs in the nucleus and cytoplasm, which in turn affects the patterns of gene expression that are important for immunological responses and the integrity of oral epithelial cells ^[57,58]^.

Within the realm of oral epithelial plasticity, HuR exerts its influence by enhancing the stability of mRNAs that provide instructions for producing crucial regulators of immune responses, epithelial cell differentiation, and tissue repair. HuR interacts with specific RNA molecules, creating complexes between RNA and proteins that protect the RNA from being broken down, ultimately leading to a longer lifespan for the RNA. The stabilization process enables the continuous and carefully controlled expression of important genes, which affects the adaptive responses of oral epithelial cells. Immunohistochemistry studies have demonstrated robust staining of HuR in the epithelium and connective tissue of inflammatory periodontal tissue, suggesting its active involvement in these regions ^[58]^. The duality of HuR’s roles is apparent in its need to safeguard the epithelial barrier against sudden inflammation or viral deterioration, while also contributing to the advancement of tumor growth. On the other hand, myeloid HuR is required to inhibit good inflammation to eliminate pathogens and decrease tumor growth ^[59]^. In addition, the involvement of HuR in the development of oral cancer has been studied in our laboratory using a mouse model that was exposed to the carcinogen 4-nitroquinoline 1-oxide (4NQO). The results indicate that HuR specifically affects T cells and the Wingless Int-1 (WNT) signaling pathway in HNSCC and regulates immunological functions, making it a potential treatment option for HNSCC cases with HuR overexpression ^[12]^.

## 7. Interaction with other RBPs

HuR frequently engages in collaboration or opposition with other RBPs to control the stability and translation of specific target mRNAs. For instance, in the context of mRNA stability, RBPs such as AUF1, BRF1, TTP, and KSRP facilitate the degradation of ARE-mRNA, which is crucial for the quick turnover of mRNAs that encode immediate-early genes. These genes are often engaged in responding to cellular stimuli ^[59]^. HuR can reverse the process of decay by binding to the same AU-rich elements (AREs), which in turn stabilizes the mRNAs and enhances their translation. In addition, the mRNA and protein stability of HuR is also controlled by other RBPs. For example, TTP and RNP C1 have the ability to affect HuR stability or protein translation, so providing an additional level of regulation over HuR’s activity in the cell ^[59]^. This regulation ensures that the levels of HuR are suitably maintained in response to the needs of the cell. It has been shown that HuR also interacts with muscle-related protein SMN in C2C12 myoblasts ^[60]^.

## 8. Post-translational modifications

The function and cellular distribution of HuR are affected by post-translational modifications, which can be influenced by interactions with other proteins. As an illustration, the process of bringing the HuR protein into the nucleus is connected to the activation of AMP-activated protein kinase (AMPK), which results in the alteration of importin α1 through both acetylation and phosphorylation. The alterations can impact HuR’s ability to move between the nucleus and cytoplasm, hence affecting its ability to control mRNA stability and translation ^[59]^, which directly influences the biological processes such as immune response and stem cell proliferation.

## 9. HuR in oral epithelium

HuR has been linked to the development of HNSCC, a common type of cancer in humans, in the oral epithelium. The overexpression of HuR contributes to the development of cancer by regulating the stability and translation of mRNAs that are involved in various cellular processes, including cell proliferation, survival, local angiogenesis, evasion of immunological detection, and metastasis ^[13]^.

## 10. Modulation of immune responses by HuR

HuR has a significant function in regulating immune responses in the oral epithelium and its plasticity. It specifically affects the expression of genes related to immune responses and inflammation ^[58]^. Multiple studies have provided insights on the function of HuR in coordinating immune responses in the oral epithelium ^[12,61]^. One component of this modulation pertains to the control of cytokine expression. For example, HuR regulates the expression of cytokines such as TNF-α and IL-6, which are important factors in inflammatory responses ^[61]^. HuR has a role in stabilizing the mRNAs of cytokines, hence increasing the inflammatory response. This reaction is crucial for combating infections and initiating tissue healing processes ^[61]^. Furthermore, HuR has been demonstrated to enhance the stability of mRNA transcripts that encode pro-inflammatory cytokines, including IL-1β and IL-8, in oral epithelial cells. This action contributes to the promotion of local inflammation ^[62]^. This indicates that HuR may play a role in starting and enhancing immune responses in the oral mucosa when exposed to microbial or inflammatory triggers.

On the other hand, HuR can potentially have a counteractive impact on inflammation in oral epithelial cells by enhancing the stability of mRNA transcripts that encode anti-inflammatory cytokines or proteins involved in regulating the immune system ^[12]^. One example is the process of HuR-mediated stability of IL-10 mRNA, which can enhance the release of this anti-inflammatory cytokine ^[59]^. IL-10 is important for reducing excessive inflammation and facilitating the healing of oral mucosa tissue ^[63]^. Furthermore, HuR has been linked to the control of immune cell recruitment and activation in the oral epithelium. HuR can affect the composition and strength of the immune response in the oral mucosa by controlling the expression of chemokines like CCL20 and CXCL1, which play a role in attracting immune cells like neutrophils and T cells ^[64]^. In addition, HuR may have a role in preserving the function and structure of the oral epithelial barrier, which is crucial for defending against microbial invasion and reducing inflammatory reactions. Research has demonstrated that HuR has the ability to control the activity of genes that produce tight junction proteins and antimicrobial peptides in the cells that make up the lining of the mouth ^[65]^. This, in turn, affects the function of the barrier and the body’s defense systems against microbes.

## 11. Therapeutic potential of targeting HuR

Due to its significant involvement in the flexibility of oral epithelial cells and the development of diseases, HuR is a very intriguing target for therapeutic intervention. Drugs have been specifically designed to target the HuR-RBP network for therapeutic use, namely in the field of cancer treatment. Below are few illustrations:

- Pyrvinium pamoate is a HuR inhibitor that has been licensed by the Food and Drug Administration (FDA). It works by blocking the movement of HuR from the nucleus to the cytoplasm. This is achieved by inhibiting the pathway involving checkpoint kinase 1 and cyclin-dependent kinase 1 and also increasing immune infiltration and combating oral tumors ^[12]^.
- MS-444 and dihydrotanshinone-I (DHTS) are compounds that function as inhibitors by specifically targeting HuR and inhibiting its ability to bind to RNA molecules. Their focus is primarily on the RRM1 and RRM2 domains of HuR, which play a critical role in its interaction with RNA ^[66]^.
- AZA-9 is a nanomolecular inhibitor that hinders the capacity of HuR to bind to RNA, thus impacting the control of genes implicated in the growth of cancer ^[66]^.
- Niclosamide, once developed as an antihelminthic medication, has been repurposed to enhance the effectiveness of cancer immunotherapy. It regulates the PD-L1 signaling pathway controlled by the RNA-binding protein HuR, which is important for the evasion of the immune system by tumors. Niclosamide can boost the death of cancer cells by T lymphocytes and improve the effectiveness of anti-PD-1 immunotherapy by preventing the movement of HuR to the cytoplasm and reducing the glycosylation of PD-L1 ^[67]^.

## 12. Conclusions

The association between HuR and other RBPs in oral epithelial cells is intricate and precisely regulated. These interactions play a vital role in regulating gene expression after transcription, influencing several processes ranging from normal cell function to the onset of illness. Gaining an understanding of these dynamics provides valuable insights into possible targets for therapeutic interventions in oral health issues. In addition to the availability of many therapies that target HuR, gaining a comprehensive understanding of HuR’s diverse activities could lead to the development of new and particularly therapeutic strategies for various diseases, such as inflammatory disorders and oral cavity tumors.

## Conflict of interest

The authors declare no conflict of interest.

## Acknowledgments

This work was supported by the National Institutes of Health (R01DE030013, R21DE032461). We want to thank Hannah Park, an undergraduate research assistant, at the University of New Mexico for her editorial assistance of the manuscript.
